# THE OBITUARY OF THE PYLORUS-PRESERVING PANCREATODUODENECTOMY

**DOI:** 10.1590/0102-6720201600020001

**Published:** 2016

**Authors:** Orlando Jorge Martins TORRES, Rodrigo Rodrigues VASQUES, Camila Cristina S. TORRES

**Affiliations:** Department of Surgery, Federal University of Maranhão, São Luiz, MA, Brazil

Pancreatoduodenectomy is the treatment of choice for patients with benign and malignant disease of pancreatic head. Classic pancreatoduodenectomy was described by Whipple originally and included distal hemigastrectomy. Pylorus-preserving pancreatoduodenectomy (pylorus-preserving) was popularized in the late 1970s for benign disease and it included full preservation of the pylorus. However, delayed gastric emptying after pylorus-preserving is a frustrating complication. Its incidence varying from 19% to 61% in previous series and it results in discomfort, prolonged length of stay and increases the risk of respiratory complications. Delayed gastric emptying contributes to increased hospital costs and decreased quality of life. There has been no evidence from prospective studies and meta-analyses to indicate the superiority of pylorus preserving in terms of quality of life or delayed gastric emptying^2^ ^,^ ^4^ ^,^ ^5^ ^,^ ^7^. 

More recently, and mostly in Japan since the late 1990s, subtotal stomach-preserving pancreatoduodenectomy (stomach-preserving) in which the pyloric ring and 2 cm of the distal stomach only is removed with preservation of about 90% of the stomach has been performed for pancreatic head disease. This surgical procedure was associated with fewer postoperative complications. After stomach-preserving, many recent studies have been carried out comparing the two techniques^2^ ^,^ ^6^ ^,^ ^8^. Subtotal stomach-preserving pancreatoduodenectomy was adopted in 2011 at the Department of Hepato-pancreatobiliary Surgery, Federal University of Maranhão, Brazil. 

Delayed gastric emptying is a very important complication and needs to be minimized in patients who undergo pancreatoduodenectomy for malignant disease. Many factors were reported in the pathophysiology of this complication after pylorus-preserving. Pylorospasm caused by operative disruption of the vagal nervous system and vascular supply with antropyloric ischemia may play a main role^2^ ^,^ ^7^ ^,^ ^9^. As for prophylactic management of pylorospasm due to denervation after pylorus-preserving, some operative technique has been described. The most common are: a) mechanical dilatation of the pylorus ring, b) pyloromyotomy, c) preservation of the right gastric artery, gastroduodenal artery and all innervation along the lesser curvature of the stomach and proximal duodenum, and d) low doses of erythromycin in the unfed period with preservation of the right gastric artery^2^ ^,^ ^7^. 

Kawai et al. in a prospective, randomized, controlled trial, reported that the overall incidence of delayed gastric emptying was 4.5% in stomach-preserving and 17.2% in pylorus preserving, a significant difference (p=0.024). Time of passage from esophagogastric junction to gastrojejunostomy or duodenojejunostomy on postoperative day 7 was significantly delayed in pylorus-preserving compared with stomach-preserving (p<0.0001)^6^. Hayashibe et al. revealed that the incidence of delayed gastric emptying in pylorus preserving was significantly higher than that in stomach-preserving (p=0.02). Days of nasogastric intubation and days until liquid diet in pylorus-preserving were significantly longer than that in stomach-preserving (p=0.002 and p=0.004, respectively)^4^. Zhou et al. reported that both overall delayed gastric emptying and clinically relevant delayed gastric emptying occurred significantly less often in the stomach-preserving group than in the pylorus-preserving (p=0.006 and p=0.013 respectively). Primary delayed gastric emptying was recorded in two patients in the stomach-preserving group and in eight in the pylorus-preserving group (p=0.041). Length of stay was significantly shorter after stomach-preserving than after pylorus-preserving (p=0.017)^9^. Fujii et al. reported that the incidence of delayed gastric emptying was significantly higher in the pylorus-preserving group than in stomach-preserving (p=0.0012). The duration of nasogastric intubation and the fasting period were significantly longer in the pylorus-preserving (p=0.0006 and p<0.0001, respectively)^1^. 

The gastric emptying requires coordination of the antrum, pylorus and duodenum. The antropyloric region is innervated by the gastric branches of the vagus nerve and by the hepatic vagal plexus. After surgery involving radical lymph node dissection in the hepatoduodenal ligament area, the motility of the antropyloric region is compromised^2^ ^,^ ^4^ ^,^ ^5^ ^,^ ^7^. 

Pylorus-preserving pancreatoduodenectomy was described for benign disease^6^. In pancreas head carcinoma, peripyloric lymph nodes metastasis was reported to be 6% to 12%. Therefore, peripyloric lymph nodes dissection is the standard procedure in order to perform R0 resection. A D2 lymph node dissection includes the area along the hepatoduodenal ligament, the common hepatic artery, the superior mesenteric artery, and peripylorus^8^. Consequently, the vagal innervation around the pyloric ring is destroyed causing dysfunction of the pylorus in pylorus-preserving. Preservation of the vagal nerve is not compatible with radical lymph node dissection of the hepatoduodenal ligament^6^ ^,^ ^8^. Kurahara et al. reported that in patients with D2 regional lymph node dissection, the pylorus-preserving group had significantly higher incidence of delayed gastric emptying (p=0.0326) and longer length of stay compared with the stomach-preserving group (p= 0.0476). On the other hand, the incidence of delayed gastric emptying and the length of stay in the two groups were comparable in patients with D1 regional lymph node dissection (p=0.3348 and p=0.1383 respectively). In this context, stomach-preserving is the most appropriate surgery involving D2 regional lymphadenectomy in order to decrease this postoperative complication^7^. 

 Poor nutritional status could result in an adverse prognosis. After pancreatoduodenectomy, approximately 12% of the body weight was lost at six months and started to recover by one year after the surgery. During this period, Fujii et al. revealed that serum total protein and albumin levels showed better recovery in the stomach-preserving group than in pylorus-preserving group, when the difference in the serum albumin levels reached statistical significance (p=0.0303)^1^. The serum total lymphocyte count was significantly higher in the stomach-preserving at one year after the surgery (p=0.0203). Serum albumin level and total lymphocyte count are important immunonutritional indicators^3^. 

The gastric outlet diameter in stomach-preserving is larger than that in pylorus-preserving, resulting in a lower incidence of delayed gastric emptying. This may have contributed to improve the oral intake followed by more favorable nutritional status. Fujii et al. reported that the diameter of the gastric outlet of the gastrojejunostomy in the stomach-preserving group was significantly larger than in the pylorus-preserving group (45±7 and 33±5 mm, respectively; p<0.0001). The possibility of adjusting the diameter of the gastric outlet may be one of the benefits of stomach-preserving. This benefit is not observed in preservation of the pyloric ring^1^ ^,^ ^3^. 

In conclusion, preservation of the pyloric ring without vagal innervation has no significance. Pylorus-preserving pancreatoduodenectomy is not compatible with R0 resection and is associated with more severe and more frequent postoperative delayed gastric emptying, increased hospital costs and decrease quality of life. The nutritional status is compromized by one year after surgery. There is no reason to maintain the pyloric ring if we want to reduce such complications. Stomach-preserving pancreatoduodenectomy should be the standard procedure for patients with pancreatic head cancer and therefore, in these circumstances the pylorus-preserving pancreatoduodenectomy is dead and buried.

## References

[1] Fujii T, Kanda M, Kodera Y (2012). Preservation of the pyloric ring has little value in surgery for
pancreatic head cancer: A comparative study comparing three surgical
procedures. Ann Surg Oncol.

[2] Hackert T, Hinz U, Hartwig W, Strobel O, Fritz S, Schneider L, Werner J, Buchler M (2013). Pylorus resection in partial pancreaticoduodenectomy impact on delayed
gastric emptying. Am J Surg.

[3] Hanna M, Gadde R, Tamariz L (2015). Delayed gastric emptying after pancreaticoduodenectomy Is subtotal
stomach preserving better or pylorus preserving?. J Gastrointest Surg.

[4] Hayashibe A, Kameyama M, Shinbo M (2007). The surgical procedure and clinical results of subtotal stomach
preserving pancreaticoduodenectomy in comparison with pylorus preserving
pancreaticoduodenectomy. J Surg Oncol.

[5] Huang W, Xiong JJ, Wan MH (2015). Meta-analysis of subtotal stomach-preserving pancreaticoduodenectomy
vs pylorus preserving pancreaticoduodenectomy. World J Gastroenterol.

[6] Kawai M, Tani M, Hirono S (2011). Pylorus ring resection reduces delayed gastric emptying in patients
undergoing pancreatoduodenectomy: A prospective.randomized.controlled trial of
pylorus resecting versus pylorus-preserving pancreatoduodenectomy. Ann Surg.

[7] Kurahara H, Takao S, Shinchi H (2010). Subtotal stomach-preserving pancreaticoduodenectomy prevents
postoperative delayed gastric emptying. J Surg Oncol.

[8] Matsumoto I, Shinzeki M, Asari S (2014). A prospective randomized comparison between pylorus- and subtotal
stomach-preserving pancreatoduodenectomy on postoperative delayed gastric emptying
occurrence and long-term nutritional status. J Surg Oncol.

[9] Zhou Y, Lin L, Wu L, Xu D, Li B (2015). A case-matched comparison and meta-analysis comparing
pylorus-resecting pancreaticoduodenectomy with pylorus-preserving
pancreaticoduodenectomy for the incidence of postoperative delayed gastric
emptying. HPB.

